# Impact of Routine Respiratory Pathogen Panel Test on Outcome of Pediatric Oncology Patients With Febrile Neutropenia

**DOI:** 10.1002/cnr2.70527

**Published:** 2026-03-30

**Authors:** Farrah Gaston, Alexander Tran, Collins Odhiambo, Mustafa Bakir

**Affiliations:** ^1^ University of Illinois College of Medicine Peoria Peoria Illinois USA; ^2^ Children's Hospital of Illinois Peoria Illinois USA

**Keywords:** antimicrobial management, febrile neutropenia, pediatric oncology, respiratory pathogen panels, viral infections

## Abstract

**Background:**

Infection is a significant cause of morbidity and mortality in pediatric oncology patients, especially during neutropenia. While bacterial infections are traditionally considered the primary cause of febrile neutropenia, the growing use of respiratory pathogen panels (RPPs) for viral detection in children has not been extensively studied for its potential beneficial impact on hospital course.

**Aim:**

This study aimed to evaluate whether routine respiratory pathogen panel (RPP) testing at admission influences clinical outcomes—including hospital length of stay, duration of neutropenia, fever duration, and PICU transfer—in pediatric oncology patients admitted with febrile neutropenia, and to identify specific respiratory pathogens independently associated with clinical course.

**Methods:**

A retrospective cohort study of pediatric oncology patients admitted for febrile neutropenia between April 2010 and December 2022 was conducted at a medium‐sized academic center. RPPs were performed on nasal swabs from 196 patients at admission, using molecular‐based polymerase chain reaction with results available within 12 h. The panel detected common respiratory viruses and atypical bacteria. Key outcomes—duration of neutropenia, fever, and length of hospital stay, transfer to pediatric intensive care unit (PICU)—were compared between patients who had positive and negative RPP results, respiratory symptoms, and individual pathogens. Viral trends before and after the COVID‐19 pandemic were also analyzed.

**Results:**

Viral infections were more prevalent than proven bacterial infections in this cohort. RPP testing, regardless of respiratory symptoms or test positivity, was not associated with significant differences in hospital length of stay or PICU admission. Parainfluenza viruses were associated with shorter neutropenia, fever duration, and hospital stay in univariate analysis. In multivariable logistic regression, detection of community coronaviruses, presence of bacterial infection, and a diagnosis of leukemia were each independently associated with increased odds of prolonged neutropenia, prolonged fever, and longer hospital length of stay.

**Conclusions:**

Routine respiratory pathogen panel testing, in the absence of RPP‐guided changes in clinical management, was not associated with reductions in hospital length of stay or overall duration of febrile neutropenia. While identification of specific viral pathogens—such as parainfluenza viruses—was associated with shorter clinical courses, these findings reflect pathogen‐specific associations rather than an effect of RPP testing itself. Consequently, this study does not provide sufficient evidence to support the routine use of respiratory pathogen panels in children admitted with febrile neutropenia under current clinical practice.

**Abbreviations**
Absolute neutrophil count: ANC
Complete blood count: CBC
Comprehensive metabolic panel: CMP
Cytomegalovirus: CMV
Length of stay: LOS
Polymerase chain reaction: PCR
Respiratory pathogen panel: RPP
Respiratory syncytial virus: RSV

## 1 Introduction

Pediatric oncology patients are particularly vulnerable to infectious complications due to chemotherapy‐induced immunosuppression, disruption of mucosal barriers, and prolonged periods of neutropenia. Febrile neutropenia remains one of the most common causes of hospitalization in children undergoing cancer treatment and is associated with significant morbidity, healthcare utilization, and cost. National data indicate that children with cancer admitted for febrile neutropenia experience median hospital stays of four to 5 days, with substantial variability depending on underlying malignancy and infectious complications [1]. Because of the potentially life‐threatening nature of bacterial sepsis in this population, standard management relies on prompt hospitalization, broad‐spectrum empiric antibiotics, and extensive diagnostic evaluation.

Although bacterial infections have traditionally been viewed as the primary concern in febrile neutropenia, advances in molecular diagnostics have demonstrated that viral pathogens are frequently detected in immunocompromised children. Prior studies using polymerase chain reaction‐based assays have reported respiratory virus detection rates ranging from 8% to nearly 60% in febrile neutropenic episodes, even in the absence of clear respiratory symptoms [2, 3, 4, 5, 6, 7, 8, 9, 10]. These findings suggest that viral infections may represent a substantial and underrecognized contributor to febrile illness in pediatric oncology patients. However, the clinical significance of viral detection in this setting remains uncertain [11], particularly with respect to outcomes such as duration of neutropenia, fever, and length of hospitalization.

Respiratory pathogen panels (RPPs) allow rapid identification of a broad range of respiratory viruses and atypical bacteria and are increasingly used in hospitalized children with suspected respiratory infections. In immunocompetent pediatric populations, RPP testing has been associated with reductions in diagnostic uncertainty and, in some settings, shorter hospital stays [12]. However, the role of RPP testing in pediatric oncology patients with febrile neutropenia is less clear. Current guidelines for pediatric febrile neutropenia emphasize prompt empiric antimicrobial therapy but do not uniformly recommend routine respiratory viral testing, in part due to limited evidence that such testing alters management or improves outcomes [13, 14]. Moreover, concerns remain regarding prolonged viral shedding, incidental detection, and the inability to safely modify antibiotic therapy in patients without evidence of marrow recovery.

Understanding the impact of viral and bacterial pathogens on clinical outcomes in pediatric oncology patients is particularly important in the context of evolving infection prevention strategies and the COVID‐19 pandemic, which has altered respiratory virus circulation patterns worldwide. Identifying whether specific respiratory pathogens are associated with more severe or prolonged clinical courses could inform risk stratification, isolation practices, and the design of future management protocols that incorporate molecular diagnostic results.

The objective of this study was to evaluate the prevalence of respiratory viral and bacterial infections detected by RPP testing in pediatric oncology patients admitted with febrile neutropenia and to assess their association with clinically relevant outcomes, including duration of neutropenia, fever, length of hospital stay, and transfer to the pediatric intensive care unit (PICU). By examining outcomes across pathogen types and pandemic periods, this study aims to clarify the clinical and epidemiologic significance of RPP testing in a vulnerable pediatric oncology population and to inform future studies evaluating RPP‐guided management strategies.

## 2 Methods

This retrospective observational cohort study analyzed the impact of viral and bacterial infections (detected via respiratory molecular testing of nasal swabs) on the duration of neutropenia, fever, hospital stays, and PICU admission in children. The study was approved by the Peoria Institutional Review Board (Approval #2059192‐2). Patient informed consent was not required because this study was a retrospective cohort analysis based on chart reviews.

### 2.1 Subjects

Five hundred twenty‐two pediatric oncology patients under 21 years, admitted for febrile neutropenia at the Children's Hospital of Illinois between April 2010 and December 2022, were included. Fever was defined as a temperature ≥ 101°F, ≥ 100.4°F for over 1 h, or two readings > 100.4°F within 12 h.

Neutropenia was defined as an absolute neutrophil count (ANC) < 500 cells/μL at admission or development of ANC < 500 cells/μL within 48 h of admission. Patients who were febrile but did not meet neutropenia criteria within 48 h were excluded. Patients with at least one of the respiratory symptoms of cough, nasal congestion, rhinorrhea, or sore throat were defined as “symptomatic.” Excluded were patients without true fever, patients who remained nonneutropenic throughout hospitalization, or those with fungal infections due to rarity and limited testing; however, no patients in this cohort were diagnosed with a fungal infection during the 1st week of admission, and therefore no exclusions were made for this reason.

### 2.2 RPP

Patients with respiratory symptoms were typically tested by RPP, while asymptomatic ones were tested for COVID‐19, influenza, and RSV (post‐COVID‐19 pandemic) or for influenza and/or RSV (pre‐COVID‐19). Specimens were collected and tested via molecular PCR for pathogens including adenovirus, coronaviruses (229E, HKU1, NL63, OC43), metapneumovirus, rhinovirus/enterovirus, influenza (A subtypes, B), parainfluenza (types 1–4), RSV, *Bordetella pertussis* , *Chlamydia pneumoniae* , *Mycoplasma pneumoniae* , and SARS‐CoV‐2. Results were available within 12 h. Routine tests included CBC, CMP, and blood cultures, while additional serologic and imaging tests were done based on clinical suspicion. Antimicrobial management was unaffected by RPP results.

Patient data included demographics, clinical outcomes, infection status (bacterial, viral, fungal, or none), respiratory symptoms, and RPP findings. Length of stay (LOS) was compared across patients with viral illnesses, respiratory symptoms, negative RPP results, bacterial infections, and pre‐ versus post‐COVID‐19 pandemic groups. Viral frequencies and types were also compared pre‐ and postpandemic, with April 1, 2020, marking the pandemic's onset.

### 2.3 Study Endpoints and Outcomes

Patient data included demographics, cancer diagnosis, respiratory symptoms, microbiologic results (RPP findings and blood culture results), and clinical outcomes. The primary endpoints were (1) duration of neutropenia (hours) and (2) hospital LOS (days). The secondary endpoints were (1) duration of fever (hours) and (2) transfer to the PICU (yes/no). Outcomes were compared across groups defined by RPP result (positive vs. negative), presence of respiratory symptoms, pathogen category (viral vs. bacterial), specific viral pathogens when sample size allowed, and by time period (pre‐COVID‐19 vs. post‐COVID‐19; April 1, 2020 as the start of the COVID‐19 period).

### 2.4 Statistical Analysis

Count data were summarized as frequencies and percentages, while continuous variables were presented as mean ± standard deviation or median (IQR), depending on the distribution. Categorical data comparisons used Pearson Chi‐Square or Fisher's Exact tests, while the Mann–Whitney U test assessed differences in fever duration, neutropenia, and LOS. Binary logistic regression identified predictors of prolonged fever (> 5 days), neutropenia (> 7 days), and LOS (> 7 days), with stepwise inclusion of significant variables. A significance threshold of 5% was applied. Data were analyzed using SPSS IBM (2024).

## 3 Results

### 3.1 Population Characteristics

**FIGURE 1:**
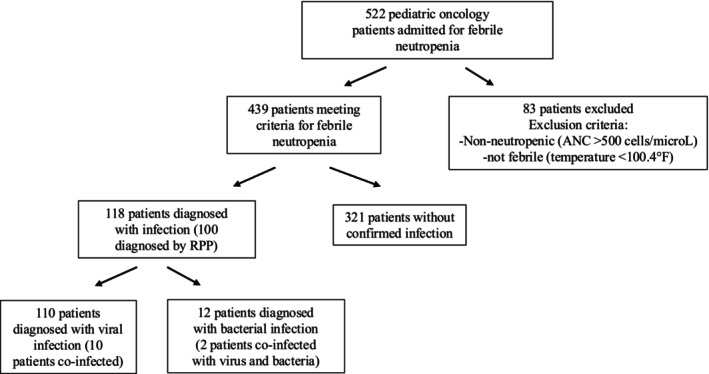
Consort diagram of patients in study.

Out of 522 hospitalized pediatric oncology patients, 439 met the criteria for febrile neutropenia (Figure 1). Table 1 summarizes their demographic and clinical characteristics.

**TABLE 1:** Demographic and clinical characteristics.

Sex, *n* (%)	Male	236 (53.8)
	Female	203 (46.2)
Mean age ± standard deviation		7.6 ± 5.5
Duration of neutropenia, *n* (%)	≤ 7 days	379 (86.3)
	> 7 days	60 (13.7)
Duration of fever, *n* (%)	≤ 5 days	409 (93.2)
	> 5 days	30 (6.8)
Respiratory symptom, *n* (%)	Yes	108 (25)
	No	331 (75)
Cancer diagnosis	Acute lymphoblastic leukemia	216 (49.2)
	Neuroblastoma	52 (11.8)
	Ewing sarcoma	34 (7.7)
	Osteosarcoma	29 (6.6)
	Wilms tumor	21 (4.9)
	Others	87 (19.8)
Length of stay, *n* (%)	≤ 7 days	371 (81.1)
	> 7 days	68 (18.9)
COVID‐19 period, *n* (%)	Pre‐COVID‐19	343 (78.1)
	Post‐COVID‐19	96 (21.9)

### 3.2 Frequency of Viral Illnesses in Febrile Neutropenia With and Without Respiratory Symptoms

Among 439 patients with febrile neutropenia, 118 (27%) were symptomatic for respiratory infection. Among those tested by RPP (*n* = 196), 99 (51%) had at least one respiratory symptom. Pathogens were detected in 118 out of 196 patients tested (60%), being a virus in 93% (*n* = 110) and bacteria in 6% (*n* = 7) (Table 2). Of those patients tested positive, 92% were symptomatic. Of note, 63 of 75 (64%) patients diagnosed with Rhino/Enterovirus were symptomatic. Other diagnostic methods included quad screen, flu A&B/RSV testing, and serology (1.9%, *n* = 8). Co‐infection with multiple viruses occurred in 10 patients, while dual viral and bacterial infections occurred in 2. No COVID‐19 infections were detected. Bacterial pathogens identified through blood cultures included *Escherichia coli* (*n* = 3), *Staphylococcus epidermidis* (*n* = 3), *Streptococcus pneumoniae* (*n* = 1), and others (details provided). Annual RPP testing ranged from 24 to 49 patients.

**TABLE 2:** Pathogens detected on RPP.

Pathogens	Total number of patients (%)^a^
Adenovirus	1 (1)
Coronavirus HKU1	4 (3)
Coronavirus NL 63	2 (2)
Coronavirus OC43	5 (4)
Metapneumovirus	3 (2)
Rhino/enterovirus	75 (61)
Influenza A	3 (2)
Influenza A, H3	2 (2)
Influenza A, 2009 H1	2 (2)
Influenza B	1 (1)
Parainfluenza virus 1	4 (3)
Parainfluenza virus 2	4 (3)
Parainfluenza virus 3	1 (1)
Parainfluenza virus 4	4 (3)
RSV	10 (8)

^a^ No cases of the following pathogens were detected: coronavirus 229E, influenza A H1, *Bordetella pertussis* , *Chlamydia pneumoniae* , *Mycoplasma pneumoniae* , *Bordetella parapertussis* (IS1001), and SARSCOV2.

### 3.3 Impact on Neutropenia, Fever, LOS, and Transfer to PICU

Viral or bacterial infections were associated with prolonged neutropenia (Table 3). Prolonged neutropenia (> 7 days) was observed in 60 patients; among these, 24 patients (40%) had a documented viral or bacterial infection (*p* = 0.012). Viral infections increased this duration significantly (35% > 7 days, *p* = 0.035), as did bacterial infections (6.7% > 7 days, *p* = 0.044). Bacterial infections also extended LOS significantly (7.4% > 7 days, *p* = 0.025). However, no significant effects on fever duration were observed. Pre‐ versus post‐COVID‐19 analyses showed no significant differences in neutropenia duration (*p* = 0.426), fever duration (*p* = 0.504), or LOS (*p* = 0.296). Univariate analysis revealed no statistically significant differences between symptomatic and asymptomatic patients tested by RPP, regardless of test result (positive or negative), in terms of fever duration, neutropenia, or LOS (*p* > 0.05 for all comparisons). Additionally, a positive RPP result did not significantly affect the rate of PICU transfer in febrile neutropenic patients (64% [9/14] vs. 9% [90/182], *p* = 0.28). Notably, two of the three noninfectious deaths occurred in patients with RPP‐detected rhino/enterovirus, suggesting possible viral carriage unrelated to mortality.

**TABLE 3:** Univariate comparison of patients in the pre‐ and post‐COVID‐19 eras and detection of different pathogens with respect to the duration of neutropenia, fever, and hospital stay.

		Duration of neutropenia (*n*, %)				Duration of fever (*n*, %)				Length of stay (*n*, %)			
		≤ 7 days	> 7 days	Total (*n*)	*p*	≤ 5 days	> 5 days	Total (*n*)	*p*	≤ 7 days	> 7 days	Total (*n*)	*p*
Pre/post‐COVID‐19	Pre‐COVID‐19	295 (77.8)	48 (80)	343	0.426	319 (78)	24 (80)	343	0.504	292 (78.7)	51 (75)	343	0.296
	Post‐COVID‐19	84 (22.2)	12 (20)	96		90 (22)	6 (20)	96		79 (21.3)	17 (25)	96	
One or more respiratory virus	No	292 (77)	39 (65)	331	0.035	311 (76)	20 (66.7)	331	0.175	283 (76.3)	48 (70.6)	331	0.197
	Yes	87 (23)	21 (35)	108		98 (24)	10 (33.3)	108		88 (23.7)	20 (29.4)	108	
One respiratory virus only	No	303 (79.9)	40 (66.7)	343	0.019	322 (78.7)	21 (70)	343	0.185	294 (79.2)	49 (72.1)	343	0.124
	Yes	76 (20.1)	20 (33.3)	96		87 (21.3)	9 (30)	96		77 (20.8)	19 (27.9)	96	
One or more nonrespiratory virus	No	377 (99.5)	60	437	0.745	407 (99.5)	30	437	0.868	370 (99.7)	67 (98.5)	437	0.286
	Yes	2 (0.5)	0 (0)	2		2 (0.5)	0 (0)	2		1 (0.3)	1 (1.5)	2	
One nonrespiratory virus only	No	377 (99.5)	60	437	0.745	407 (99.5)	30	437	0.868	370 (99.7)	67 (98.5)	437	0.286
	Yes	2 (0.5)	0 (0)	2		2 (0.5)	0 (0)	2		1 (0.3)	1 (1.5)	2	
Any virus (both respiratory and others)	No	290 (76.5)	39 (65)	329	0.043	309 (75.6)	20 (66.7)	329	0.191	282 (76)	47 (69.1)	329	0.146
	Yes	89 (23.5)	21 (35)	110		100 (24.4)	10 (33.3)	110		89 (24)	21 (30.9)	110	
At least one bacteria present	No	371 (97.9)	56 (93.3)	427	0.044	399 (97.6)	28 (93.3)	427	0.195	364 (98.1)	63 (92.6)	427	0.025
	Yes	8 (2.1)	4 (6.7)	12		10 (2.4)	2 (6.7)	12		7 (1.9)	5 (7.4)	12	
Either virus or bacteria is present	No	285 (75.2)	36 (60)	321	0.012	303 (74.1)	18 (60)	321	0.075	277 (74.7)	44 (64.7)	321	0.062
	Yes	94 (24.8)	24 (40)	118		106 (25.9)	12 (40)	118		94 (25.3)	24 (35.3)	118	
Any coronavirus present	No	370 (97.6)	58 (96.7)	428	0.457	399 (97.6)	29 (96.7)	428	0.545	361 (97)	67 (98.5)	428	0.471

### 3.4 Effect of RPP and Pathogen Type on Recovery

Comparisons between patients with positive RPP results, negative RPP results, and no RPP testing revealed no significant differences in neutropenia duration, fever duration, or LOS. However, parainfluenza viruses were linked to shorter neutropenia duration and LOS in univariate analysis (Table 4). Rhinovirus/enterovirus, RSV, and coronaviruses did not significantly impact outcomes. Data for influenza, adenovirus, and metapneumovirus were insufficient for analysis. Logistic regression identified coronaviruses, bacterial infections, and leukemia as key factors associated with prolonged neutropenia, fever, and LOS (Table 5).

**TABLE 4:** Univariate comparisons of duration of neutropenia, duration of fever, and length of stay by pathogen.

Pathogen detected, *n*	Variable	Test result	Median (hours/days)	IQR	*p* ^a^
Rhino/enterovirus (*n* = 75)	Duration of neutropenia (hours)	Detected	96	71	0.05
		Not detected	73	59.5	
	Duration of fever (hours)	Detected	24	33.5	0.369
		Not detected	24	34.8	
	Length of stay (days)	Detected	4	4	0.410
		Not detected	3	3	
RSV (*n* = 10)	Duration of neutropenia (hours)	Detected	84.5	83	0.803
		Not detected	73	71	
	Duration of fever (hours)	Detected	33.3	74	0.466
		Not detected	24	33	
	Length of stay (days)	Detected	3.5	4	0.962
		Not detected	4	3	
Parainfluenza viruses (*n* = 13)	Duration of neutropenia (hours)	Detected	49	48	0.017
		Not detected	73	71	
	Duration of fever (hours)	Detected	15	19	0.006
		Not detected	24	38.5	
	Length of stay (days)	Detected	2	2	0.001
		Not detected	4	4	
Coronaviruses (*n* = 11)	Duration of neutropenia (hours)	Detected	168	143	0.110
		Not detected	73	71	
	Duration of fever (hours)	Detected	21	115	0.852
		Not detected	24	33.5	
	Length of stay (days)	Detected	4	6	0.458
		Not detected	4	3	

^a^ Mann–Whitney U test.

**TABLE 5:** Results of conditional model selection for logistic regression.

	Duration of neutropenia (< 7 days vs. ≥ 7 days)	Duration of fever (< 5 days vs. ≥ 5 days)	Length of stay (< 7 days vs. ≥ 7 days)
	Odds Ratio (95% Confidence Interval)		
Any coronavirus present	8.80 (2.59–29.92)	5.18 (1.26–21.33)	4.06 (1.21–13.67)
At least one bacteria present	4.19 (1.19–14.83)	7.64 (1.80–32.43)	8.53 (2.43–29.91)
Leukemia	1.26 (0.42–3.29)	8.52 (1.25–6.95)	0.879 (0.49–1.55)

### 3.5 Impact of COVID‐19 Pandemic on Respiratory Virus Detection

Among patients tested using RPPs, fewer respiratory viruses were detected in the post‐COVID‐19 period compared to the pre‐COVID‐19 period (42% vs. 55.9%, *p* = 0.044); this comparison was based on RPP‐tested at admissions, with 343 patients (78.1%) tested prepandemic and 96 patients (21.9%) tested postpandemic. However, the types of viruses detected did not differ significantly.

## 4 Discussion

Our study highlights the predominance of respiratory viral infections among pediatric oncology patients hospitalized for febrile neutropenia. Among patients with an identifiable infection, the majority were diagnosed via RPP testing, with 93.2% (*n* = 110) yielding positive RPP results. The infection rate in this cohort (26.9%) aligns with previous studies showing high viral detection rates in febrile neutropenic episodes [7, 8, 9]. Our data aligns with reported viral detection rates as much as 60% using sensitive PCR‐based methods, like those used here [7, 8, 9] For instance, one study found respiratory viruses in 52% of febrile neutropenic children with negative bacterial cultures [10].

With the increasing use of RPPs over the past decade as a diagnostic tool for children admitted with respiratory symptoms, previous reports in the immunocompromised pediatric population may be outdated. Current RPPs test for more than 20 of the most common respiratory pathogens and have been shown to decrease the length of stay in nonneutropenic patients with viral illnesses [12]. However, the safety of stopping empiric antibacterial therapy in patients with positive respiratory viral tests in patients with negative blood cultures but no evidence of marrow recovery is not well known. A recent prospective randomized study involving 139 children with cancer reported that there are no significant differences in resolution rates, along with shorter durations of therapy and fever and efficacy of early discontinuation of antimicrobial therapy in children with neutropenic fever diagnosed with a respiratory virus via RPP [15].

Current recommendations for the workup of febrile neutropenia by the Infectious Diseases Society of America (IDSA) and American Society of Clinical Oncology (ASCO) Guideline Panel include a complete blood count (CBC), complete metabolic panel (CMP), blood cultures from all lumens, and a viral panel in adults [13]. However, updated ASCO guidelines for the management of fever and neutropenia in pediatric patients with cancer do not specifically recommend routine respiratory viral panel testing as part of the initial workup for febrile neutropenia [14] since the utility of RVP testing in altering clinical outcomes such as LOS, discontinuation of antibiotics, or ICU admission has not been definitively established.

This study demonstrated some associations between infectious etiology and clinical outcomes, that is, viral or bacterial infections were linked to longer neutropenia and fever duration. Bacterial infections specifically correlated with extended neutropenia and hospital stays. Viral infections in general had no significant impact on duration of fever and LOS regardless of existence of respiratory symptoms, however, prolonged the duration of neutropenia that might be explained by possible bone marrow suppression. Parainfluenza viruses showed potential associations with shorter hospitalizations, though these results were confounded. Coronaviruses were associated with prolonged neutropenia and hospital stays. Previous research has shown that immunocompromised patients with coronavirus infections face higher risks of severe outcomes [16, 17]. Although we have not included the duration of antimicrobial treatment in our analyses, febrile neutropenia protocol in our institution was not modified based on the result of RPP. These findings do not provide convincing evidence supporting routine use of RPP at the admission of children with cancer and febrile neutropenia. Identifying viral pathogens could reduce unnecessary antibiotic use and resistance, particularly in cases with influenza or SARS‐CoV‐2 where antiviral therapies exist.

In our study, rhino/enterovirus (61%), parainfluenza (10%), coronaviruses (9%), RSV (8%), and influenza (8%) were the most commonly detected viruses. No COVID‐19 cases were observed, because infected patients did not meet fever or neutropenia criteria. Although some detected viruses may represent prolonged shedding rather than active infection, their identification can influence clinical management and infection prevention practices. For example, 40% of our cases positive for Rhino/Enterovirus were asymptomatic, however, RPP test provided opportunity of barrier precautions.

Patients with leukemia had longer fevers in our study, consistent with prior studies showing hematologic malignancies as predictors of prolonged hospitalizations [18, 19]. The broader timeframe of this study (2010–2022) also revealed more detected pathogens before the COVID‐19 pandemic.

Limitations of our study include the retrospective design, variability in RPP usage, and seasonal variations in respiratory virus prevalence. Despite these limitations, our findings align with updated ASCO guidelines for the management of fever and neutropenia in pediatric patients with cancer [13] that do not specifically recommend routine use of RPP as part of the initial workup for febrile neutropenia. Although fewer respiratory viruses were detected in the post‐COVID‐19 period compared to the prepandemic period, this analysis was limited to patients who underwent RPP testing, and fewer patients were tested in the postpandemic era, which may have contributed to the observed difference. Another important limitation of this study is that the febrile neutropenia management protocol at our institution was not altered based on RPP results. Because antimicrobial management was not altered based on RPP results per institutional protocol, this study was not designed to evaluate whether identification of specific pathogens via RPP testing could influence antimicrobial selection, duration, or de‐escalation. As a result, patients with positive and negative RPP findings received similar antimicrobial therapy and were managed using the same discharge criteria. Under these circumstances, differences in hospital LOS or duration of fever and neutropenia would not necessarily be expected based solely on RPP positivity. Therefore, our findings should not be interpreted as evidence against the potential utility of RPP testing when used to guide clinical decision‐making. Rather, they reflect outcomes under a standardized treatment approach in which RPP results were not used to modify care. Future prospective studies evaluating RPP‐guided management strategies, including early antibiotic de‐escalation, isolation practices, or discharge planning are needed to better define the clinical utility of RPP testing in pediatric oncology patients with febrile neutropenia.

## 5 Conclusion

In pediatric oncology patients admitted with febrile neutropenia, RPP testing was not associated with differences in hospital LOS, transfer to the PICU, or overall duration of febrile neutropenia, likely reflecting standardized management protocols that were not influenced by RPP results. However, identification of specific viral pathogens provided prognostic insight, as certain viruses were associated with shorter clinical courses. For pediatric oncology patients, these findings suggest that while routine RPP testing alone may not alter acute management under current practice, pathogen identification may aid in risk stratification, infection control measures, and future development of RPP‐guided management strategies. Prospective studies incorporating clinical decision‐making based on RPP results are needed to define the role of RPP testing in improving outcomes for this vulnerable population.

## Author Contributions

**Farrah Gaston:** conceptualization, investigation, formal analysis, resources, writing – original draft, writing – review and editing, visualization. **Alexander Tran:** investigation, resources, writing – original draft, writing – review and editing. **Collins Odhiambo:** formal analysis, visualization. **Mustafa Bakir:** conceptualization, writing – original draft, writing – review and editing, supervision. All authors had full access to the data in the study and take responsibility for the integrity of the data and the accuracy of the data analysis.

## Funding

The authors have nothing to report.

## Ethics Statement

This study was performed in line with the principles of the Declaration of Helsinki. Approval was granted by the Peoria Institutional Review Board Ethics Committee (Date: August 1, 2023/No: 2059192‐3).

## Consent

Authors have provided the statement in rebuttal letter. Authors have published the preprint. Please request to include the statement in the manuscript.

## Conflicts of Interest

The authors declare no conflicts of interest.

## Data Availability

The data that support the findings of this study are available from the corresponding author upon reasonable request.
